# The Inositol-3-Phosphate Synthase Biosynthetic Enzyme Has Distinct Catalytic and Metabolic Roles

**DOI:** 10.1128/MCB.00039-16

**Published:** 2016-05-02

**Authors:** Anna D. Frej, Jonathan Clark, Caroline I. Le Roy, Sergio Lilla, Peter A. Thomason, Grant P. Otto, Grant Churchill, Robert H. Insall, Sandrine P. Claus, Phillip Hawkins, Len Stephens, Robin S. B. Williams

**Affiliations:** a Centre for Biomedical Sciences, School of Biological Sciences, Royal Holloway University of London, Egham, Surrey, United Kingdom; b The Babraham Institute, Cambridge, Cambridgeshire, United Kingdom; c Department of Food and Nutritional Sciences, The University of Reading, Reading, Berkshire, United Kingdom; d Cancer Research UK Beatson Institute, Bearsden, Glasgow, United Kingdom; e Department of Pharmacology, University of Oxford, Oxford, Oxfordshire, United Kingdom

## Abstract

Inositol levels, maintained by the biosynthetic enzyme inositol-3-phosphate synthase (Ino1), are altered in a range of disorders, including bipolar disorder and Alzheimer's disease. To date, most inositol studies have focused on the molecular and cellular effects of inositol depletion without considering Ino1 levels. Here we employ a simple eukaryote, Dictyostelium discoideum, to demonstrate distinct effects of loss of Ino1 and inositol depletion. We show that loss of Ino1 results in an inositol auxotrophy that can be rescued only partially by exogenous inositol. Removal of inositol supplementation from the *ino1*^−^ mutant resulted in a rapid 56% reduction in inositol levels, triggering the induction of autophagy, reduced cytokinesis, and substrate adhesion. Inositol depletion also caused a dramatic generalized decrease in phosphoinositide levels that was rescued by inositol supplementation. However, loss of Ino1 triggered broad metabolic changes consistent with the induction of a catabolic state that was not rescued by inositol supplementation. These data suggest a metabolic role for Ino1 that is independent of inositol biosynthesis. To characterize this role, an Ino1 binding partner containing SEL1L1 domains (Q54IX5) and having homology to mammalian macromolecular complex adaptor proteins was identified. Our findings therefore identify a new role for Ino1, independent of inositol biosynthesis, with broad effects on cell metabolism.

## INTRODUCTION

A stereoisomer of inositol, *myo*-inositol, is present in a variety of cell types and is obtained from the following three major sources: *de novo* synthesis from glucose-6-phosphate, sequential dephosphorylation of phosphoinositides, and membrane transport from extracellular fluid (1). Disruption of inositol homeostasis has been associated with a number of illnesses, including bipolar disorder (2–5), Alzheimer's disease (6–9), bulimia (10), metabolic syndrome (11), diabetes (12, 13), and epilepsy (14). Understanding the cellular and metabolic changes resulting from inositol depletion will provide insight into the mechanisms underlying these diseases.

**FIG 1:**
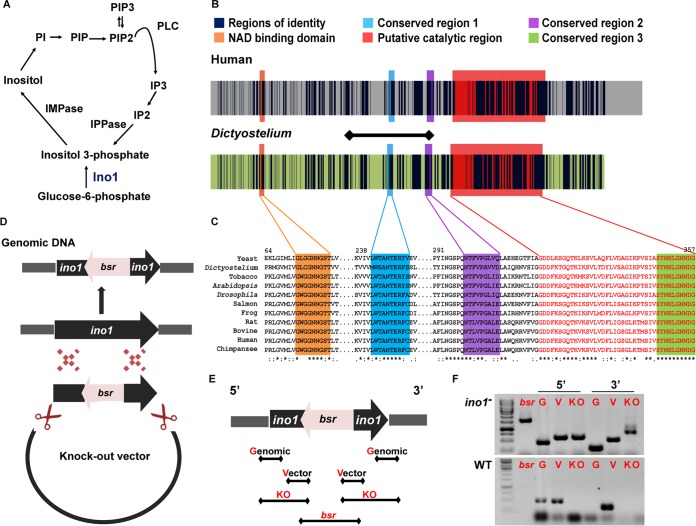
Inositol signaling and conservation of the Ino1 protein in Dictyostelium and humans. (A) Inositol metabolism. Ino1 converts glucose-6-phosphate to inositol-3-phosphate, which is a rate-limiting step in inositol production. (B) Sequence homology between the human (accession no. Q9NPH2-1) and Dictyostelium (accession no. Q54N49) Ino1 proteins is present throughout the protein sequences. Identical amino acids are shown in dark blue. The NAD binding and catalytic domains are among the four regions that are highly conserved in eukaryotic Ino1 proteins, i.e., for the human protein GWGGNNG (orange), LWTANTERY (blue), SYNHLGNNDG (green), and NGSPQNTFVPGL (purple). The tetramerization domain, containing a putative catalytic site (with the conserved amino acid residues SYNHLGNNDG), is shown in red. The amino acids that were ablated in Dictyostelium Ino1 are shown by the horizontal line. (C) Alignment of the conserved regions of Ino1 proteins from various species. Asterisks show identity, colons show high conservation levels, and dots show low conservation levels. (D) Schematic representation of the strategy used to prepare the *ino1* knockout construct. 5′ and 3′ regions of the *ino1* gene were cloned into a knockout vector at positions flanking the blasticidin resistance gene (*bsr*), and the knockout cassette was transformed into Dictyostelium cells, where homologous recombination deleted a portion of the *ino1* gene and disrupted the open reading frame. (E) PCR screening strategy to identify *ino1*^−^ mutants, showing primer locations for genomic and vector controls, the diagnostic knockout products (KO), and the area spanning the inserted *bsr* gene present in the *ino1*^−^ knockout. (F) PCR results showing the ablation of part of the *ino1* gene in the *ino1*^−^ mutant in comparison to wild-type cells. Ino1, inositol-3-phosphate synthase; IMPase, inositol monophosphatase; IPPase, inositol polyphosphate 1-phosphatase; IP2, inositol bisphosphate; IP3, inositol trisphosphate; PLC, phospholipase C; PI, phosphatidylinositol; PIP, phosphatidylinositol phosphate; PIP2, phosphatidylinositol bisphosphate; PIP3, phosphatidylinositol trisphosphate; *bsr*, blasticidin resistance gene; G, genomic control; V, vector control; KO, knockout; 5′, region corresponding to the transcription initiation site of the *ino1* gene; 3′, region corresponding to the transcription termination site of the *ino1* gene.

Inositol-3-phosphate synthase (Ino1; EC 5.5.1.4) is crucial for the *de novo* biosynthesis of inositol, as it is an isomerase that converts glucose-6-phosphate to inositol-3-phosphate, which is then dephosphorylated to inositol (15) (Fig. 1A). Inositol is an essential precursor of a large family of phosphoinositides (16), with one of these, phosphoinositide 4,5 bisphosphate (PIP2), used in the production of inositol phosphates. These molecules are important for a range of cellular functions, including motility (17), activation of signal transduction pathways (18), membrane trafficking and vesicular transport (1), protein secretion, and transcriptional regulation (19). Despite these broad functions, few studies have compared the physiological effects of reducing inositol levels and reducing Ino1 levels; therefore, it remains unclear if these two activities have distinct roles.

Dictyostelium discoideum is a single-celled eukaryote found in forest soils, where it survives by consuming bacteria. Dictyostelium is used as a research model in a variety of disciplines, including biomedicine. We previously employed Dictyostelium in a 3Rs approach (animal reduction, replacement, and refinement) for biomedical research to investigate the effects of epilepsy treatments on modulating phosphoinositide signaling and seizure control (14, 20) and the effects of bipolar disorder treatments on the levels of inositol phosphates (5, 21). These findings were successfully translated to mammalian disease models (14, 21, 22). Dictyostelium was also used to identify targets for compounds involved in bitter tastant detection (23, 24) and conserved roles of homologues of human proteins (23, 25) and to investigate mitochondrial disease (26), Huntington's disease (27), and centrosomal organization and function (28, 29). These studies suggest that Dictyostelium can inform our understanding of cellular function relevant to human disease.

Dictyostelium was previously employed to investigate the role of Ino1 in cell function (30), where insertional mutagenesis of *ino1* produced an inositol-auxotrophic phenotype with a concomitant decrease in inositol trisphosphate. Here we independently deleted a key region of the *ino1* gene in an isogenic cell line and found that growth of the *ino1*^−^ mutant can be rescued only partially by exogenous inositol, suggesting a nonbiosynthetic role for the protein. We further show that the previously described “inositol-less death” is likely to lead to an upregulation of autophagy, loss of substrate adhesion, and reduced cytokinesis resulting from inositol depletion. We also show that inositol depletion leads to a generalized reduction of phosphoinositide levels, without gross changes in metabolic profile. Surprisingly, we show that the greatest metabolic change is caused by a loss of Ino1, not by inositol depletion *per se*, since broad metabolic changes are not rescued by exogenous inositol, suggesting distinct effects of Ino1 loss and inositol depletion on cellular function. Finally, we identified a range of potential Ino1 binding partners and confirmed direct Ino1 binding to a protein with mammalian homologues that serve as adaptors involved in the attachment to macromolecular complexes, providing a potential link to regulating inositol-independent cellular functions.

## MATERIALS AND METHODS

### Materials.

Axenic, HL5, and LoFlo media were purchased from ForMedium Co. Ltd. (Hunstanton, United Kingdom). All restriction enzymes and first-strand cDNA synthesis kits were purchased from Fermentas (St. Leon-Rot, Germany). Tris-HCl, NaCl, EDTA, 4′,6-diamidino-2-phenylindole (DAPI), cyclic AMP (cAMP), potassium phosphate monobasic (KH_2_PO_4_), potassium phosphate dibasic (K_2_HPO_4_), *myo*-inositol, and methanol were purchased from Sigma (Dorset, United Kingdom). High Pure RNA isolation kits were purchased from Roche (Welwyn Garden City, United Kingdom). Penicillin-streptomycin and blasticidin were purchased from Life Technologies (Gibco, United Kingdom). DNase-free DNase I kits were purchased from Ambion (Austin, TX). Anti-red fluorescent protein (anti-RFP) antibody (5F8), anti-green fluorescent protein (anti-GFP) antibody (3H9), and RFP-Trap or GFP-Trap agarose beads (ChromoTek) were purchased from ChromoTek GmbH (Planegg-Martinsried, Germany). The anti-FLAG M2 antibody (F3165) was obtained from Sigma (Dorset, United Kingdom).

### Cell culture, strains, and plasmids.

All Dictyostelium axenic strains were grown at 22°C in axenic medium containing 100 μg/ml penicillin and 100 μg/ml streptomycin. Dictyostelium transformants with a disrupted *ino1* gene were cultured in axenic medium with 10 μg/ml blasticidin and 500 μM *myo*-inositol.

Knockout constructs were created by amplifying 5′ and 3′ fragments within the *ino1* gene from genomic DNA of the Dictyostelium discoideum axenic 2 (AX2) strain by PCR. The 5′ and 3′ PCR fragments were cloned into the pLPBLP expression vector (31) by using BamHI-PstI and NcoI-KpnI restriction sites, respectively. The knockout cassette was transformed into wild-type (AX2) cells, and transformants were selected in axenic medium containing blasticidin (10 μg/ml). Independent clones of transformants resistant to blasticidin were screened for homologous integration by PCR. Loss of gene transcription was confirmed by reverse transcription-PCR. For this purpose, RNAs were extracted from the independent clones by use of a High Pure RNA isolation kit according to the manufacturer's instructions. Contaminating DNA was removed by use of a DNase-free DNase I kit, followed by cDNA synthesis using a first-strand cDNA synthesis kit with 1 μg of RNA per sample. The cDNA was analyzed by PCR to confirm the loss of gene transcription (using primers GCTGCAAATCAAAAGGATCGTGCC and AAGGTGTTTTGTGGTGAACCATTGATG).

The Ino1-RFP overexpression construct was prepared using the full-length *ino1* (gene ID DDB_G0285505) open reading frame. The gene was amplified from genomic DNA by use of EcoRI and BamHI flanking restriction sites (using primers GAGCGAATTCATGTCAGCACAAATGTTTGAATC and TATGGATCCTAATCTTTGTTCTAATAACATG). The PCR products were cloned into an mRFPmars expression vector (389-2) under the control of the actin15 promoter (courtesy of Annette Müller-Taubenberger [32, 33]). Constructs were transformed into the *ino1*^−^ cell line by electroporation and selected for neomycin resistance (10 μg/ml). Expression of Ino1-RFP was confirmed by fluorescence microscopy and Western blot analysis using anti-RFP antibodies. *ino1* gene expression was confirmed by reverse transcription-PCR, using the same method as that described for generating an *ino1* knockout cell line.

### Development assays and cell image acquisition.

Filter assays were used to develop Dictyostelium cells as described previously (5). Briefly, cells grown in the presence or absence (24 h) of inositol (500 μM) were harvested during log-phase growth, and cells (1 × 10^7^/ml) were plated on a 47-mm nitrocellulose filter (Millipore, Watford, United Kingdom). Filters were incubated for 24 h at 22°C prior to imaging.

### Substrate adhesion assay.

*ino1*^−^ or Ino1-RFP-expressing *ino1*^−^ cells grown in axenic medium in the presence of inositol (500 μM) were plated in 6-well plates, and the medium was replaced with axenic medium in the absence or presence of inositol (500 μM). After 24 h, the medium was gently removed with an aspirator to dispose of the nonadherent cells. Fresh medium was added and cells immediately resuspended and counted, and the process was repeated for later time points.

### Chemotaxis, autophagy, and cytokinesis assays.

Chemotaxis assays were carried out by using a Dunn chamber (Hawksley, Sussex, United Kingdom) as previously described (34). Images were recorded every 15 s over a 15-min period. Autophagy was measured in *ino1*^−^ cells transformed with the *atg8-GFP* construct (35; www.dictybase.org). Cells were grown in axenic medium with shaking for 72 h (no-inositol conditions had inositol removed for 24 h prior to the experiment), with 16 h of incubation in LoFlo medium to reduce the background autofluorescence. Cytokinesis defects were measured in cells cultured in shaking suspension for 72 h, with inositol removed 24 h before the start of the assay where indicated, and cells were fixed with 100% methanol at −20°C for 15 min prior to labeling with DAPI.

### Immunoprecipitation.

Initial coimmunoprecipitations were performed with an *ino1*^−^ cell line constitutively expressing the *ino1-RFP* gene; *ino1*^−^ cells constitutively expressing the *mRFPmars* gene on its own were used as a control (for 2 of 3 repeats), or wild-type (AX2) cell lysate was used as a control. The presence of Ino1-RFP and RFP was confirmed by Western blotting with anti-RFP antibody. The gel was stained with Coomassie blue, the protein bands specific to Ino1-RFP (and absent in the control) were evaluated by mass spectrometry, and the data were analyzed using Scaffold3 software.

An *ino1*^−^ cell line cotransformed with the *ino1-RFP* construct and *FLAG-gpmA*, *FLAG-pefB*, or *FLAG-Q54IX5* was used for coimmunoprecipitation with anti-RFP-coated beads to examine the direct interaction between Ino1 and these proteins. Ino1-GpmA and Ino1-Q54IX5 interactions were detected by Western blotting with anti-RFP and anti-FLAG antibodies. An *ino1*^−^ cell line cotransformed with the *ino1-RFP* construct and either *GFP-gpmA* or *GFP-Q54IX5* was used for coimmunoprecipitation with anti-GFP-coated beads to confirm an interaction between Ino1 and these proteins; *ino1*^−^ cells coexpressing *mRFPmars* and either *GFP-gpmA* or *GFP-Q54IX5* were used as a control for these experiments. The Ino1-Q54IX5 interaction was confirmed by Western blot analysis with anti-GFP and anti-RFP antibodies.

Cells (3 × 10^8^ per experiment) were washed with phosphate buffer, treated with 2.5 mM caffeine for 20 min with shaking, and lysed (0.5% NP-40, 40 mM Tris-HCl, 20 mM NaCl, 5 mM EGTA, 5 mM EDTA, 10 mM dithiothreitol [DTT], 1 mM phenylmethylsulfonyl fluoride [PMSF], and 2× protease and 2× phosphatase inhibitor cocktail [Roche]) on ice, and the lysate was incubated with RFP-Trap or GFP-Trap agarose beads per the manufacturer's instructions. Briefly, the lysate was incubated with the beads for 1 h at 4°C and then collected and washed twice (10 mM Tris-HCl, 150 mM NaCl, 0.5 mM EDTA, 1 mM PMSF, and 2× protease and 2× phosphatase inhibitor cocktail). The nonbound fraction was collected after this step. Immunocomplexes were dissociated from the beads by incubation at 70°C for 10 min in 4× TruPAGE LDS sample buffer (Sigma) and then collected by centrifugation (the bound fraction) prior to SDS-PAGE using either a Sigma TruPAGE or Bio-Rad precast gel system. The presence of protein was detected with anti-GFP or anti-RFP antibody or with a monoclonal anti-FLAG M2 antibody and recorded using an Odyssey Sa infrared imaging system.

### NMR spectrometry.

Freeze-dried cell pellets were resuspended in 1 ml of water-methanol (1:2) and vortexed for polar metabolite extraction. Samples were then centrifuged at 2,400 × *g* for 5 min, and supernatants were kept for drying using a vacuum concentrator for 4.5 h at 45°C. Once dried, each sample was resuspended in 80 μl of phosphate buffer [in 90% D_2_O and 0.05% sodium 3-(trimethylsilyl) propionate-2,2,3,3-d_4_ (TSP) as a ^1^H nuclear magnetic resonance (NMR) reference], and 50 μl of the solution was transferred to a 1.7-mm capillary NMR tube. Spectra were acquired at 300 K on a Bruker Avance DRX 700-MHz NMR spectrometer (Bruker Biopsin, Rheinstetten, Germany) operating at 700.19 MHz and equipped with a CryoProbe from the same manufacturer. All spectra were acquired using a 1-dimensional nuclear Overhauser effect spectroscopy (NOESY) pulse sequence (recycle delay-90°-t1-90°-tm-90°-acquire free induction decay [FID]), with water suppression applied during a recycle delay (RD) of 2 s, a mixing time (tm) of 100 ms, and a 90° pulse set at 7.70 μs. For each spectrum, 512 scans were accumulated over a spectral width of 9,803.9 Hz, and all FID values were multiplied by a broadening line function of 0.3 Hz prior to Fourier transformation. All spectra were manually phased, baseline corrected, and calibrated to the TSP standard at δ 0.000 by using MestReNova software (version 10.0.1; Mestrelab Research S.L., Spain).

### Phospholipid analysis.

Glycerophospholipid levels were analyzed by mass spectroscopy as previously described (36).

## RESULTS

### Ino1 protein is conserved from Dictyostelium to humans.

To investigate the role of the Dictyostelium Ino1 protein, we first compared the Dictyostelium (accession no. Q54N49) and human (accession no. Q9NPH2-1) protein sequences (Fig. 1B and C). The proteins share 59% sequence identity throughout their length, have similar sizes, and show common conserved NAD binding and catalytic domains (Fig. 1B) that are present in Ino1 proteins from species across distant biological kingdoms (Fig. 1C), suggesting a highly conserved catalytic role of Ino1 throughout evolution and supporting the use of Dictyostelium to analyze Ino1 function.

### An *ino1*^−^ strain is an inositol auxotroph.

**FIG 2:**
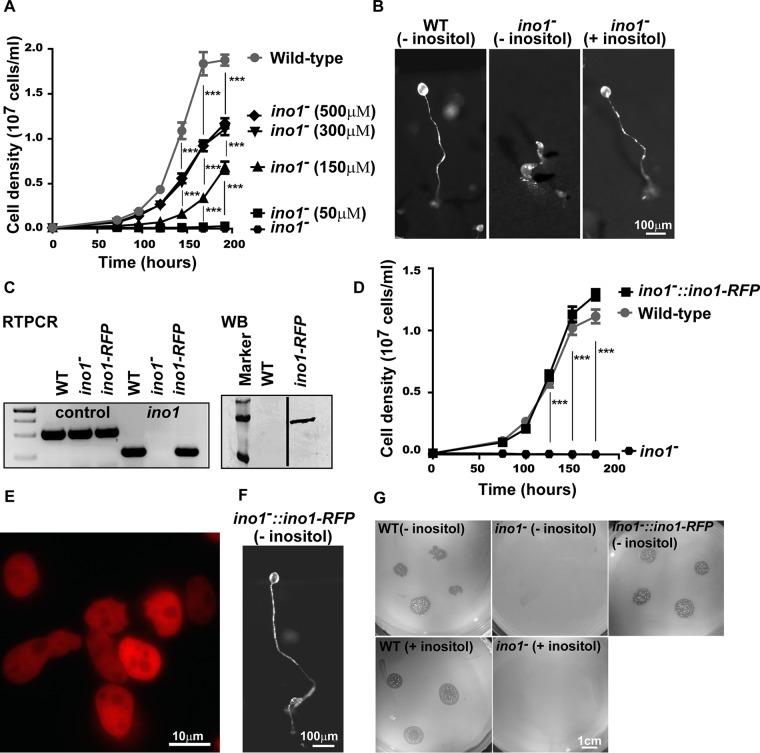
Ablating *ino1* in Dictyostelium causes inositol auxotrophy. (A) Dictyostelium cells grown in liquid medium showed rapid growth up to stationary phase (at 168 h). Ablation of *ino1* blocked cell growth in the absence of exogenous inositol, with only partial restoration of wild-type growth by the addition of either 300 μM or 500 μM inositol. (B) During starvation, wild-type (WT) Dictyostelium formed fruiting bodies without inositol pretreatment. Under the same conditions, *ino1*^−^ cells were unable to form fruiting bodies. Fruiting body formation in *ino1*^−^ cells was restored when the cells were grown with inositol supplementation prior to the assay. (C) Expression of *ino1-RFP* in Dictyostelium *ino1*^−^ cells was confirmed by reverse transcription-PCR (RTPCR) with an *ig7* gene control and by Western blot analysis (WB) to show the full-length protein (with a ladder). (D to F) The full-length protein restored the growth rate (D), was present in the cytosol (E), and restored development in the absence of exogenous inositol (F). (G) *ino1*^−^ cells were unable to grow on agar plates seeded with bacteria, and expression of *ino1-RFP* in these cells restored bacterial growth. For panels A and D, statistical significance was determined by two-way analysis of variance (ANOVA) with the Bonferroni posttest. ***, *P* < 0.001. Error bars represent standard errors of the means (SEM) for 3 experimental repeats.

To analyze the effects of Ino1 loss and inositol depletion on Dictyostelium cell growth and development, we ablated 19% of the *ino1* coding sequence, including two regions encoding highly conserved amino acid motifs, by homologous integration of a knockout cassette (Fig. 1B to F). The resultant *ino1*^−^ cells were unable to grow in liquid medium without inositol supplementation at concentrations above 50 μM (Fig. 2A), consistent with results shown previously (30). However, unlike the results of the previous study, inositol supplementation did not fully restore the *ino1*^−^ growth rate to that of the wild type, with the cells reaching a maximal level of growth at 300 μM and with higher concentrations not increasing growth.

In Dictyostelium, starvation triggers cell differentiation and morphogenesis to form spore-bearing fruiting bodies. We thus investigated the effect of Ino1 loss, with and without inositol supplementation, on multicellular development. Wild-type and *ino1*^−^ cells were starved on nitrocellulose filters for 24 h, and fruiting body morphology was recorded (Fig. 2B). *ino1*^−^ cells grown in the absence of inositol for 24 h prior to nutrient deprivation were able to aggregate but formed aberrant fruiting bodies (Fig. 2B), a phenotype not observed for *ino1*^−^ cells in an earlier report (30); however, inositol supplementation (500 μM) prior to the assay enabled *ino1*^−^ cells to produce mature fruiting bodies with wild-type morphology.

Both growth and development phenotypes were due to a lack of the Ino1 protein. This was shown by expression of Ino1 linked to a C-terminal RFP tag in *ino1*^−^ cells, which was localized in the cytosol and restored wild-type growth and development (Fig. 2D to G). Interestingly, since exogenous inositol did not fully restore the wild-type growth rate in *ino1*^−^ cells, but Ino1-RFP did, it is likely that cells require the Ino1 protein for normal growth. *ino1*^−^ cells were also unable to grow on a bacterial lawn (Fig. 2G), as reported previously (28), even with inositol supplementation. These results confirm a vital role for inositol in Dictyostelium growth and development, consistent with that shown in a variety of organisms throughout the kingdoms of life (37).

### Ino1 loss triggers inositol depletion.

**FIG 3:**
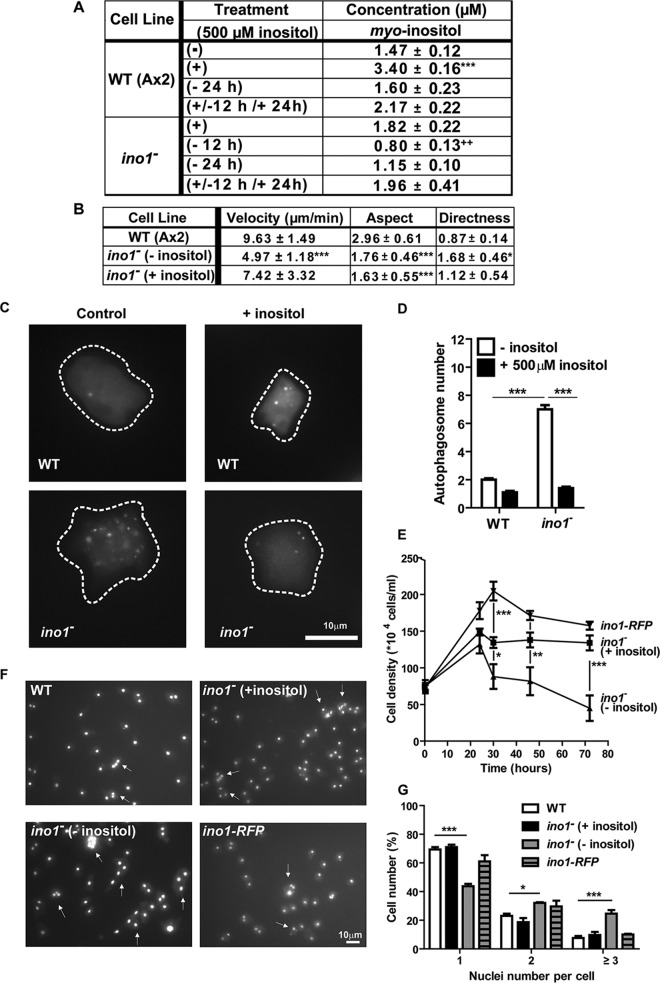
Inositol depletion causes a change in velocity and cell shape, an activation of autophagy, a loss in cell-substrate adhesion, and a reduction in cytokinesis in Dictyostelium *ino1*^−^ cells. (A) Levels of *myo*-inositol, as analyzed by NMR spectroscopy, in wild-type and *ino1*^−^ cells grown with (+; 500 μM) or without (−) exogenous inositol for 12 or 24 h or following inositol reintroduction (+/−). Data shown are means ± SEM. Inositol levels were reduced in the *ino1*^−^ mutant following inositol depletion for 12 and 24 h and were restored to basal levels following reintroduction for 12 h. (B) Average velocity, aspect, and persistence of aggregation-competent *ino1*^−^ cells (grown with [+] or without [−] 500 μM inositol for 24 h prior to imaging) or wild-type cells during chemotaxis toward cAMP. Velocity shows the distance traveled by cells over time. Aspect refers to the roundness of cells (1 = perfectly round). Directness is a ratio of the distance traveled by a cell to the total direct distance, where 1 represents a straight line. (C and D) Autophagosomes were visualized in wild-type and *ino1*^−^ cells expressing Atg8-GFP (C) and quantified in the absence of inositol (24 h) (control) or presence of inositol (D). (E) Cell adhesion was monitored in wild-type and *ino1*^−^ cells and in *ino1*^−^ cells expressing *ino1-RFP* in the presence (500 μM) or absence of inositol for at least 24 h. (F and G) Cytokinesis was examined in wild-type and *ino1*^−^ cells and in *ino1*^−^ cells expressing *ino1-RFP* by using the DAPI nuclear stain to label cell nuclei (F), and the number of nuclei per cell was quantified (G). For panels A, B, D, and E, statistical significance was determined by the unpaired two-tailed *t* test, with each condition compared separately to wild-type cells (− inositol) (A, B, and D) and *ino1*^−^ cells (+ inositol) (D). For panels E and G, statistical significance was determined by 2-way ANOVA with the Bonferroni posttest. *, *P* < 0.05; **, *P* < 0.01; ***, *P* < 0.001. Error bars represent SEM. For panel A, there were 4 experimental repeats. For panel B, ≥20 cells were analyzed per condition in 3 experimental repeats. For panel D, 117 cells were analyzed per condition in 3 experimental repeats. For panel E, there were 3 experimental repeats. For panel G, 386 cells were analyzed per condition in 3 experimental repeats.

We then quantified inositol levels in the *ino1*^−^ and wild-type cells in the presence or absence of added inositol by performing NMR spectroscopy (Fig. 3A). Wild-type cells grown in unsupplemented medium contained 1.5 ± 0.1 μM inositol, and this increased significantly, to 3.4 ± 0.1 μM, following inositol supplementation (*P* < 0.0001), and returned to baseline following removal of inositol (Fig. 3A). In contrast, *ino1*^−^ cells grown with inositol supplementation had an intermediate level of inositol (1.8 ± 0.1 μM) that significantly decreased, to 0.8 ± 0.1 μM, following removal of exogenous inositol for 12 h (*P* = 0.0013). A reduced level was maintained following 24 h of starvation (1.2 ± 0.1 μM), and the level returned to 2.0 ± 0.1 μM following reintroduction of inositol (Fig. 3A). These data confirm that in *ino1*^−^ cells, inositol was depleted following withdrawal of exogenous inositol, and this trend is consistent with that reported earlier (30). In addition, these data suggest that the *ino1*^−^ mutant supplemented with inositol has intracellular inositol levels similar to those in wild-type cells (without supplementation) and that differences between these cell types are likely to arise from an absence of the enzyme, enabling a range of experiments to provide new insights into the distinct cell and metabolic changes caused by inositol depletion and loss of Ino1.

### Ino1 loss causes a pleiotropic phenotype.

We first investigated potential changes in cell movement during chemotaxis toward cAMP (Fig. 3B). In these experiments, wild-type cells showed a velocity of 9.6 ± 1.5 μm/min, with an elongated shape (aspect) and a tendency for single-directional movement (directness) of 0.87 ± 0.14. Loss of Ino1 plus inositol supplementation caused a significant loss of the elongated shape, suggesting an Ino1-dependent change. In contrast, inositol depletion in *ino1*^−^ cells significantly reduced the cell speed, while the loss of shape that was also observed for Ino1 deletion was retained, and triggered increased persistence. These data suggest distinct effects specific to Ino1 loss (loss of cell shape) and to inositol depletion (loss of velocity).

We then examined the mechanism leading to the block in cell growth caused by a loss of Ino1 in the absence of exogenous inositol, previously termed ‘“inositol-less death” (38). Since autophagy can lead to cell death in response to cell stress or nutrient depletion (39), we tested whether inositol depletion triggered an autophagic response. In Dictyostelium, formation of autophagosomes can be visualized by the incorporation of fluorescently tagged autophagy-related protein 8 (Atg8) into autophagosomal membranes (35). The *ino1*^−^ strain grown in the absence of inositol for 24 h showed a 4-fold increase in the number of autophagosomes per cell compared to the wild-type strain (Fig. 3C and D). These data suggest that inositol depletion triggered an autophagic response in *ino1*^−^ cells.

We also examined the effects of Ino1 loss and inositol depletion on substrate attachment and cytokinesis. To assess changes in cell adhesion, the number of cells attached to plates was quantified for up to 72 h after the removal of exogenous inositol from the *ino1*^−^ mutant. In the presence of inositol (500 μM), *ino1*^−^ cells proliferated for up to 24 h and remained adherent (Fig. 3E). Upon removal of exogenous inositol, the *ino1*^−^ cell number decreased to 88.5% of inositol-supplemented cells after 24 h and to 33.5% after 72 h. *ino1*^−^ cells expressing *ino1-RFP* did not lose adhesion in the absence of exogenous inositol. We then assessed cytokinesis by comparing the numbers of nuclei per cell for the *ino1*^−^ and wild-type strains, in the presence of inositol or following inositol depletion, by using the DAPI nuclear stain (Fig. 3F and G) (40). In these experiments, *ino1*^−^ cells showed a significant (*P* < 0.001) increase in the number of nuclei following inositol depletion compared to that for the wild-type strain. Under inositol depletion conditions, 24.7% of the *ino1*^−^ cells accumulated ≥3 nuclei, compared to 7.7% of the wild-type cells. This effect was rescued by growing *ino1*^−^ cells in the presence of inositol (500 μM) (9.7% of cells accumulated ≥3 nuclei) or by overexpressing *ino1*-RFP (Fig. 3F and G) (10% of cells accumulated ≥3 nuclei). These data suggest that inositol depletion leads to an increase in autophagy, a loss of cell-substrate adhesion, and a reduction in cytokinesis but that the loss of Ino1 *per se* does not trigger these responses.

### Inositol depletion regulates phospholipid levels.

**FIG 4:**
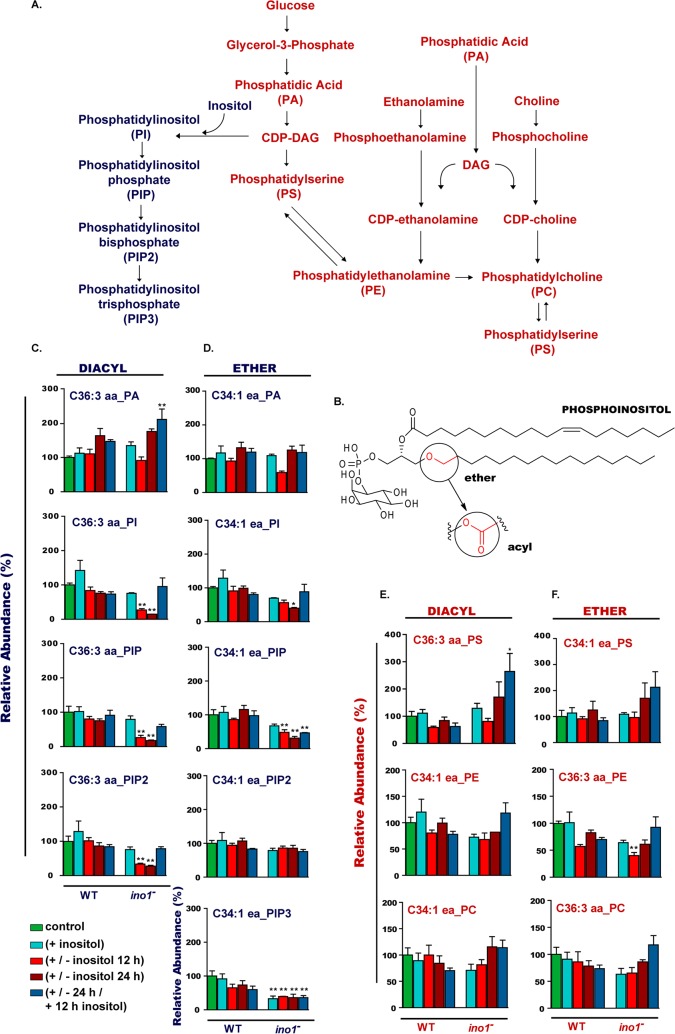
Inositol depletion affects phosphoinositide levels in Dictyostelium. (A) Metabolic pathway depicting phospholipid production from phosphatidic acid (PA) as an example. (B) Structure of phosphoinositol, showing diacyl or ether/acyl fatty acid linkages to a glycerol backbone and an inositol head group. (C to F) To monitor phospholipids in wild-type and *ino1*^−^ mutant cells, cells were grown in the presence of inositol (500 μM; +), with inositol followed by inositol withdrawal (for 12 or 24 h; +/−), or with inositol added after a 24-h depletion period (500 μM for 12 h; +/−/+). The controls were cells without inositol supplementation. The levels of ether/acyl (C34:1 ea) or diacyl (C36:3 aa) phospholipids are shown as percentages relative to phospholipid levels present in the wild-type strain grown in the absence of inositol. Inositol depletion reduced the levels of diacyl PI, PIP, and PIP2 phosphoinositides; the level of diacyl PIP3 was undetectable, and the levels of ether/acyl PIP and PIP3 were reduced. Statistical analysis was carried out by comparing two groups at a time: wild-type (+ inositol) versus *ino1*^−^ (+ inositol) cells, wild-type (+ inositol) versus *ino1*^−^ (+/− inositol 12 h) cells, wild-type (+ inositol) versus *ino1*^−^ (+/− inositol 24 h) cells, and wild-type (+ inositol) versus *ino1*^−^ (+/− 24 h/+ 12 h inositol) cells. Comparisons were done by the unpaired two-tailed *t* test to illustrate the significance of changes due to the loss of the Ino1 protein. *, *P* < 0.05; **, *P* < 0.01; ***, *P* < 0.001. Error bars represent SEM for 3 experimental repeats.

Since inositol is a precursor to a family of inositol phospholipids (Fig. 4A and B), we examined changes in phospholipid levels due to the loss of Ino1 and as a result of inositol depletion. In Dictyostelium, the following two types of phospholipids are present: diacyl phospholipids, containing two acyl linkages to the glycerol backbone; and the recently reported ether/acyl phospholipids, containing a glycerol backbone linked to a fatty alcohol at position 1 (36) (Fig. 4B). We quantified the levels of both phospholipid species in wild-type and *ino1*^−^ cells grown in the presence and absence of inositol (Fig. 4C to F). Separation of distinct phospholipid species was limited to those of different molecular weights. We first examined levels of the phospholipid precursor phosphatidic acid (PA), which comprises a glycerol backbone and two fatty acid tails. Both diacyl-linked and ether-linked PA levels decreased during early inositol depletion in *ino1*^−^ cells (Fig. 4C and D). Phosphatidylinositol (PI), which is formed by the addition of the inositol head group to PA, decreased following inositol depletion (in *ino1*^−^ cells), with the greatest reduction seen in diacyl-linked PI (Fig. 4C and D). A similar effect was seen for diacyl phosphatidylinositol monophosphate (PIP) (Fig. 4C and D). Surprisingly, inositol depletion induced a reduction in diacyl phosphatidylinositol bisphosphate (PIP2) but not in ether/acyl PIP2 (Fig. 4C and D). For phosphatidylinositol trisphosphate (PIP3), only ether/acyl PIP3 was detectable in *ino1*^−^ cells, and the level was reduced compared to that in wild-type cells, independent of the exogenous inositol supply (Fig. 4D). The reintroduction of inositol for 12 h after 24 h of starvation restored the levels of the majority of ether/acyl and diacyl phospholipids. These data suggest that the pool of diacyl phospholipids is more sensitive to inositol depletion than ether/acyl species and that cellular ether/acyl PIP2 levels are maintained under these conditions.

Since a reduction in inositol synthesis attenuates the production of phosphoinositides and causes a transient reduction of PA, we then monitored changes in other phospholipids during inositol depletion and rescue. No change in phosphatidylserine (PS) was seen in wild-type cells under any condition tested; however, diacyl phosphatidylserine levels were significantly increased in the *ino1*^−^ cells after 24 h of inositol starvation followed by inositol resupplementation (Fig. 4E and F). Phosphatidylcholine levels did not change significantly in wild-type or *ino1*^−^ cells under any condition (Fig. 4E and F), while the ether/acyl phosphatidylethanolamine level was decreased in *ino1*^−^ cells after 12 h of inositol starvation (Fig. 4F).

### Ino1 loss causes a shift to catabolic metabolism.

**FIG 5:**
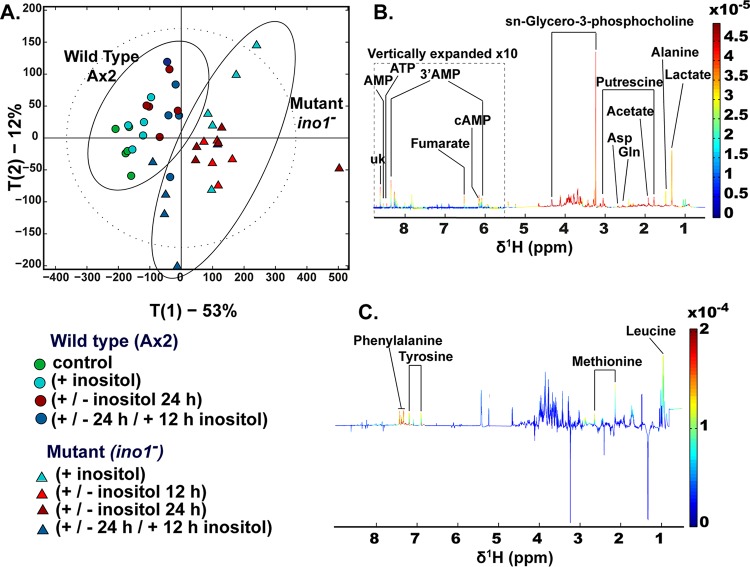
Comparison of metabolic profiles of Dictyostelium following Ino1 loss and inositol depletion. To monitor metabolic profiles in the wild type and the *ino1*^−^ mutant, cells were grown in the presence of inositol (500 μM; +), with inositol followed by inositol withdrawal (12 or 24 h; +/−), or with inositol added after a 24-h depletion period (500 μM for 12 h; +/−/+). The controls were cells without inositol supplementation. (A) Distribution of metabolic variations between cell type and *myo*-inositol exposure were assessed by principal component analysis (PCA) of data generated from the ^1^H-NMR spectra of the Dictyostelium metabolic fingerprints. The main source of variation (53%) was driven by the mutation, while inositol depletion accounted for approximately 12% of the metabolic variation in this data set. (B) Loading plot associated with PC1 (red peaks pointing upwards are positively associated with PC1, while those pointing downwards are negatively associated with PC1). (C) Loading plot associated with PC2. A total of 5 experimental repeats were performed.

We next investigated the metabolic consequences of both the loss of Ino1 and inositol depletion by using wild-type and *ino1*^−^ cells grown in the presence and absence of inositol (Fig. 5). Both ablation of *ino1* and inositol treatment induced specific metabolic changes. Principal component (PC) analysis of metabolic profiles suggested that the greatest metabolic change was observed between the wild-type and *ino1*^−^ cells independently of exogenous inositol provision (Fig. 5A and B), where *ino1* ablation accounted for 53% of the total variance, as observed in PC1. The mutation resulted in increases in amino acids and compounds related to amino acid breakdown (alanine, aspartate, isoleucine, lysine, methionine, GABA, and putrescine), in energy-related metabolites (fumarate and lactate), in phosphorylated adenosine derivatives (5′AMP, 3′AMP, ATP, and cAMP), and in *sn*-glycero-3-phosphocholine (GPC), a potent osmolyte (Fig. 5B). In contrast, inositol treatment accounted for only 12% of the variance between the metabolic profiles of wild-type and *ino1*^−^ cells, as observed in PC2 (Fig. 5A and C). In *ino1*^−^ cells, inositol treatment resulted in increased amino acid levels (leucine, methionine, and tyrosine). These data suggest a dominant role for the presence of the Ino1 protein (rather than inositol levels) in metabolic regulation (Fig. 5).

**FIG 6:**
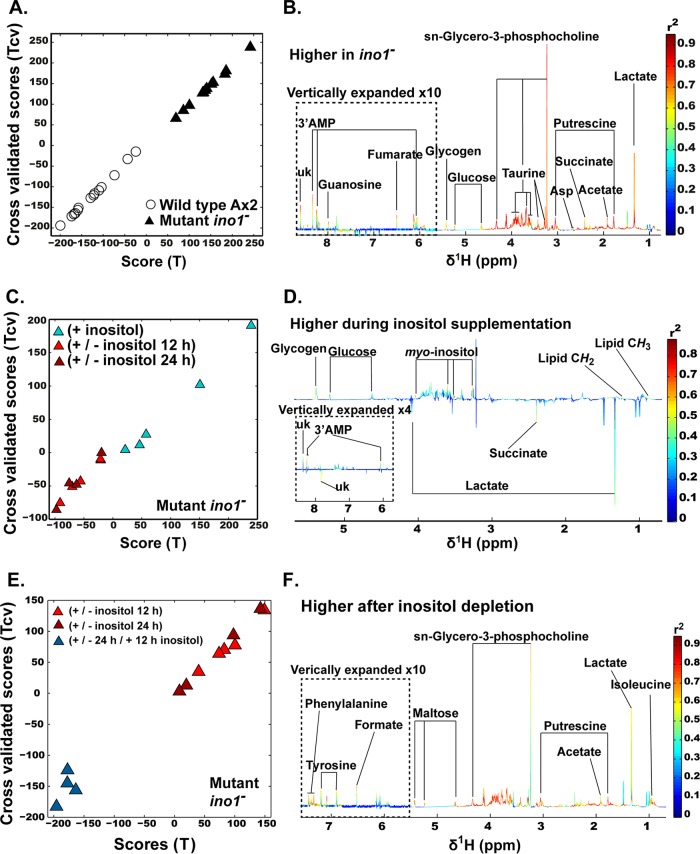
Metabolic profile analysis of the *ino1*^−^ mutant. Cells were grown in the presence of inositol (500 μM; +), with inositol followed by inositol withdrawal (12 or 24 h; +/−), or with inositol added after a 24-h depletion period (500 μM for 12 h; +/−/+). (A and B) Metabolic changes induced by *ino1* ablation. Orthogonal projection to latent structure discriminant analysis (O-PLS DA) was used to determine the specific impact of the mutation on cell metabolism. (A) Plot of the scores against the cross-validated scores generated by O-PLS DA (*R*^2^*Y* = 0.89, *Q*^2^*Y* = 0.88, and *P* value for 500 random permutations = 0.002) by using the ^1^H-NMR spectra of the Dictyostelium wild-type and *ino1*^−^ cells (except for the +/− 24 h/+ 12 h inositol exposure) as a matrix of independent variables and using mutation as a predictor. (B) Loading plot of the O-PLS DA model (peaks in red indicate increased metabolite levels in response to the mutation). (C and D) Effect of inositol treatment on the metabolism of the *ino1*^−^ mutant. (C) Plot of the scores against the cross-validated scores generated by O-PLS DA (*R*^2^*Y* = 0.67, *Q*^2^*Y =* 0.51, and *P* value for 500 permutations = 0.002) by using the ^1^H-NMR spectra of the *ino1*^−^ cells (−12 h and −24 h inositol versus + inositol) as a matrix of independent variables and using depletion of *myo*-inositol as a predictor. (D) Loading plot of the O-PLS DA model (peaks in red indicate increased metabolite levels in response to the presence of inositol). (E and F) Reintroduction of *myo*-inositol postdeprivation induces a metabolic shift. (E) Plot of the scores against the cross-validated scores generated by O-PLS DA (*R*^2^*Y* = 0.90, *Q*^2^*Y* = 0.86, and *P* value for 500 permutations = 0.002) by using the ^1^H-NMR spectra of the *ino1*^−^ cells (−12 h and −24 h inositol versus +/−/+ inositol) as a matrix of independent variables and using *myo*-inositol reintroduction as a predictor. (F) Loading plot of the O-PLS DA model (peaks in red indicate increased metabolite levels in response to the depletion of *myo*-inositol). A total of 4 experimental repeats were performed.

The absence of Ino1 caused a major shift in metabolic profile, and we therefore specifically examined changes caused by Ino1 loss (Fig. 6A and B). This analysis showed changes in many of the metabolic products found in the initial PC analysis. In contrast to a loss of Ino1, inositol depletion caused limited changes to metabolic profiles. Here we specifically compared *ino1*^−^ cells grown in the presence or absence of inositol (12- and 24-h treatments were combined because they resulted in similar metabolic changes and inositol levels) (Fig. 6C and D) to show that inositol supplementation led to an increase of inositol and lipids, consistent with the phosphoinositide analysis (Fig. 4). Interestingly, reintroduction of inositol for 12 h after 24 h of inositol depletion changed the metabolic profile of *ino1*^−^ cells (Fig. 6E and F).

**FIG 7:**
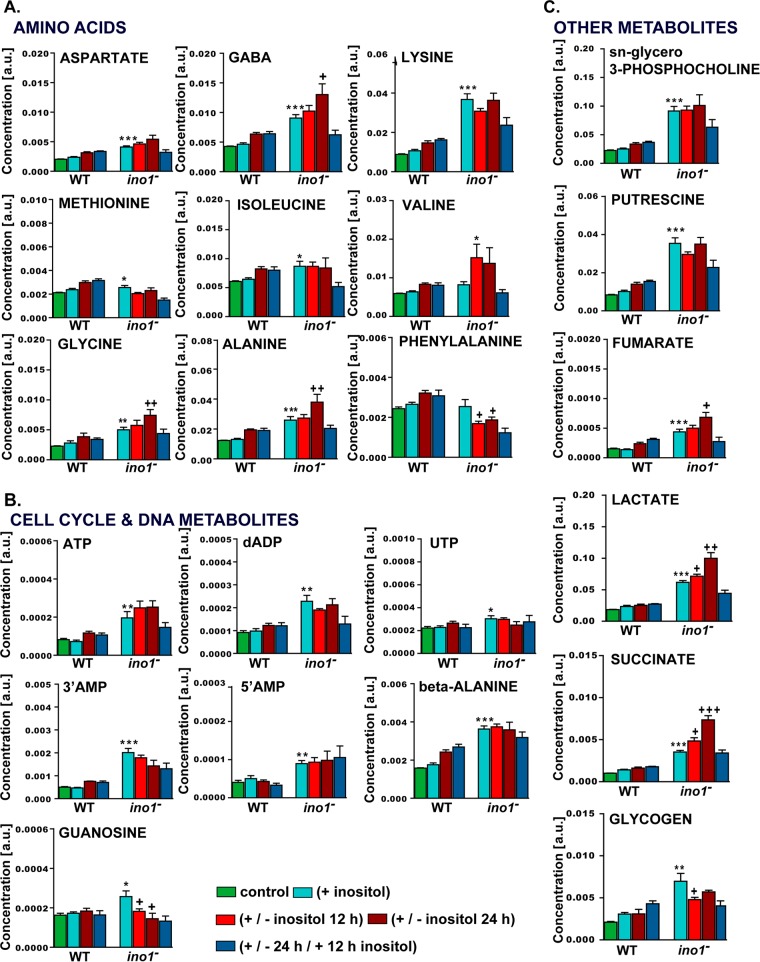
Levels of metabolites in wild-type and *ino1*^−^ cells grown under various inositol conditions. Metabolite levels measured by NMR spectroscopy were quantified using MATLAB and plotted to illustrate changes observed in wild-type and *ino1*^−^ cells for amino acids (A), cell cycle- and DNA-related metabolites (B), and other metabolites (C). The controls were cells without inositol supplementation. Statistical analysis was carried out for wild-type (AX2) (+ inositol) versus *ino1*^−^ (+ inositol) cells by the unpaired two-tailed *t* test to illustrate the significance of changes due to the loss of the Ino1 protein. *, *P* < 0.05; **, *P* < 0.01; ***, *P* < 0.001. A separate unpaired two-tailed *t* test analysis was used to compare *ino1*^−^ (+ inositol) versus *ino1*^−^ (− inositol 12 h) cells and *ino1*^−^ (+ inositol) versus *ino1*^−^ (− inositol 24 h) cells. +, *P* < 0.05; ++, *P* < 0.01; +++, *P* < 0.001. Error bars represent SEM for 5 experimental repeats per sample per condition. a.u., arbitrary units.

Supervised analysis was then used to specifically evaluate the impact of Ino1 loss on cell metabolism (Fig. 6 and 7). This approach suggested that a loss of Ino1 was associated with significant increases in some amino acids (alanine, aspartate, glycine, GABA, isoleucine, lysine, and methionine) and in metabolites associated with regulation of the cell cycle and DNA metabolism (guanosine, ATP, deoxy-ADP, 5′AMP, 3′AMP, UTP, and β-alanine, a biomarker of the degradation of pyrimidines [41]). Putrescine was also significantly increased, consistent with a reduction in cell proliferation, as previously demonstrated for Dictyostelium (42). An increase in lactate was also observed, which suggests an increase in the (NADH + H^+^)/NAD^+^ ratio that stimulates the activity of the lactate dehydrogenase. An increase in (NADH + H^+^)/NAD^+^ ratio would simultaneously inhibit the citrate synthase and slow down the Krebs cycle, resulting in an accumulation of some intermediates. This is consistent with the accumulation of acetate, derived from the spontaneous hydrolysis of oxaloacetate, and of fumarate and succinate, two other intermediates of the Krebs cycle. Finally, the GPC level was greatly increased, suggesting that the lack of Ino1 was compensated for by the production of a strong osmolyte. The increased (NADH + H^+^)/NAD^+^ ratio is a signature of catabolic reactions. Together, these data suggest that the loss of Ino1 shifts cells into a catabolic state and further support the autophagic phenotype of *ino1*^−^ mutants, even when supplemented with inositol.

Supervised analysis was also used to evaluate the impact of inositol depletion on individual metabolites (Fig. 6 and 7). This approach suggested that inositol depletion resulted in changes in some amino acids (increases in alanine, GABA, glycine, and valine and a decrease in phenylalanine), increases in lactate, fumarate, and succinate, and decreases in 3′AMP, guanosine, and glycogen. No effect on the metabolic profile was shown to be due to the selection antibiotic (blasticidin) for the *ino1*^−^ cells (orthogonal projection to latent structure [O-PLS] model parameters: R^2^*Y* = 0.18 and *Q*^2^*Y* = 0). Although we observed that the mutants were already in a catabolic state, the addition of inositol tended to moderate this metabolic phenotype, since indicators of anabolism (glycogen and lipids) were higher in cells supplemented with inositol, while those not supplemented were associated with markers of catabolism (i.e., lactate and succinate). However, the absence of Ino1 rather than inositol depletion triggered broader metabolic changes.

### Mutation of an Ino1 catalytic residue reduces growth independently of exogenous inositol.

**FIG 8:**
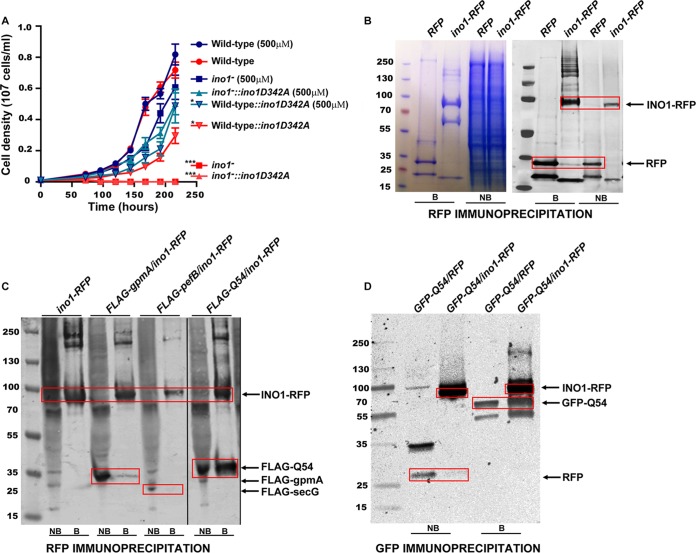
Noncatalytic role for Ino1 in Dictyostelium. (A) An Ino1-RFP protein with an aspartic acid-to-alanine substitution (*ino1D342A*) in a highly conserved region of a catalytic domain was overexpressed in wild-type and *ino1*^−^ cells. In the *ino1*^−^ cells, the mutated protein was unable to rescue the *ino1*^−^ inositol auxotrophy, consistent with a catalytically inactive protein. In the wild-type cells, expression of the mutant protein significantly decreased growth, while the addition of exogenous inositol partially rescued this phenotype. Statistical analysis was carried out for each individual condition compared to wild-type (AX2) cells by the unpaired two-tailed *t* test. *, *P* < 0.05; ***, *P* < 0.001. Error bars represent SEM for 3 experimental repeats. (B) Coimmunoprecipitation of the Ino1-RFP protein (or an RFP-only control) expressed in *ino1*^−^ cells by use of anti-RFP-coated beads. Both bound (B) and nonbound (NB) fractions are shown. Proteins in SDS-PAGE gels were visualized following Coomassie staining (left) and analyzed by Western blotting with an anti-RFP antibody (right). Bands specific to Ino1-RFP (and absent from the RFP control) were analyzed by mass spectrometry to identify potential Ino1 binding partners. (C) FLAG-tagged potential interacting proteins GpmA, PefB, and Q541X5 were investigated by immunoprecipitation using Ino1-RFP and anti-RFP-coated beads, followed by Western blotting with anti-RFP and anti-FLAG antibodies. (D) An Ino1-Q54IX5 interaction was confirmed by immunoprecipitation of the GFP-Q54IX5 protein with anti-GFP-coated beads in the presence of Ino1-RFP (or RFP only) and Western blot analysis with anti-RFP and anti-GFP antibodies.

To investigate a role for Ino1 that is independent of catalytic activity, we expressed a mutated Ino1 protein lacking a key catalytic aspartic acid (D342A) that is conserved throughout the tree of life (37). Wild-type cells expressing this construct showed strongly reduced growth in either the presence or absence of inositol (500 μM) (Fig. 8A), suggesting a dominant negative effect of the protein. *ino1*^−^ cells expressing this construct retained the inositol-auxotrophic phenotype, confirming a lack of catalytic activity of the mutated protein, but additionally showed strongly reduced growth in the presence of inositol (500 μM).

### Ino1 binds a possible macromolecular complex linker protein.

To investigate a mechanism for Ino1 in regulating cell function independently of catalytic activity, Ino1 binding partners were isolated by coimmunoprecipitation. Ino1-RFP was expressed in *ino1*^−^ cells, bound to anti-RFP antibody-coated agarose beads, and purified by coimmunoprecipitation, followed by SDS-PAGE separation and mass spectrometry analysis (Fig. 8B). This approach identified 104 potential binding partners from three independent experiments, and these were divided into six major groups, namely, actin-related, immunity- and stress-related, metabolism, nucleic acid-related (translation, transcription, regulation of gene expression, and DNA recombination), protein catabolism, and modification and transport proteins, as well as others, encompassing signal transduction, ATP hydrolysis, and proton transport (including V-type proton ATPase catalytic subunits A and B) (see the supplemental material). We extended our analysis for three potential Ino1 binding proteins: GpmA, a phosphoglycerate mutase that catalyzes the production of 2,3-bisphospho-d-glycerate (2,3BPG), which was found to accumulate in *ino1*^−^ cells starved of inositol (30); PefB, a penta-EF hand domain-containing protein linked to neurodegenerative and lysosomal diseases (43, 44); and Q54IX5, an uncharacterized protein with three Sel1-like repeats which was present in all three independent immunoprecipitation experiments (Fig. 8C and D). These proteins tagged with a FLAG epitope were coexpressed in cells with Ino1-RFP, and Ino1-RFP was immunoprecipitated from cell lysates by use of an RFP antibody linked to agarose beads. The bound protein fractions were then analyzed for the presence of each FLAG-tagged protein, which demonstrated that GpmA-FLAG bound Ino1-RFP weakly, whereas Q54IX5-FLAG bound strongly to Ino1-RFP (Fig. 8C). The Q541X5-Ino1 interaction was confirmed by using the reverse approach, where Q54IX5-GFP was coexpressed with Ino1-RFP and immunoprecipitated with a GFP antibody linked to agarose beads; coimmunoprecipitated Ino1-RFP was detected by Western blotting with an RFP antibody (Fig. 8D). These approaches confirmed that Q54IX5 binds strongly to Ino1.

## DISCUSSION

Inositol and inositol-containing compounds are vital cellular components, and a range of studies have identified pleiotropic effects of inositol depletion on cell function but have not considered complications due to altered levels of the biosynthetic enzyme Ino1. To distinguish between the effects of inositol depletion and a loss of Ino1 on cell function and metabolism, we ablated the inositol biosynthetic enzyme Ino1 in Dictyostelium and compared wild-type cells and cells without Ino1 in the presence and absence of inositol. Loss of Ino1 produced an inositol-auxotrophic phenotype during growth and blocked development, confirming the results of an earlier Dictyostelium study (30) and results for diverse organisms, ranging from Saccharomyces cerevisiae (45) to mice (46), demonstrating the essential conserved role of inositol in cellular function. We showed that the *myo*-inositol levels decreased by 36 to 56% in the *ino1*^−^ mutant (depending upon starvation time) and returned to predepletion levels following inositol replenishment. This inositol depletion response is consistent with an obligate role for inositol production catalyzed by Ino1. We showed that inositol depletion resulting from *ino1* ablation blocks development, reduces cell velocity, upregulates autophagy, and inhibits cytokinesis, consistent with a range of studies in other systems (19, 30, 47–49) and confirming the validity of this model for studying Ino1 function. All of these phenotypes, except growth and cell shape, are rescued by provision of exogenous inositol and are thus likely to be due to inositol depletion rather than the loss of Ino1.

Dysregulation of inositol levels has been reported in a wide range of biomedical and clinical studies, both relating to disease conditions and as a result of medicinal treatment, although few studies have addressed specific changes in Ino1 protein levels. A range of structurally independent bipolar disorder drugs, including carbamazepine, valproate, and lithium, act via an inositol depletion mechanism (5) and induce autophagy *in vitro* and *in vivo* (50, 51) to promote survival by recycling cellular components (47, 49). Altered inositol levels have also been demonstrated in patient studies of bipolar disorder (52), major depressive disorder (53), and schizophrenia (54). For these reasons, modulating inositol levels was proposed as a therapy for the treatment of bipolar disorder (55), depression, and panic disorders (56). In addition, Ino1 activity and protein levels are elevated in postmortem brains of Alzheimer's patients (57), although studies showed pathologically lowered inositol levels and mitochondrial dysfunction in mouse models of Alzheimer's disease (8) that could be linked to autophagy (58). However, no distinction was made in these studies between altered inositol levels and altered Ino1 levels. In the present study, we separated the effects caused by altered Ino1 levels and inositol depletion to provide a unique approach to monitor cellular and metabolic changes related to inositol levels.

Since phosphoinositide production is the first step of inositol incorporation into cell signaling, we examined the effects of a loss of Ino1 and inositol depletion on this family of chemicals, analyzing both diacyl-linked and ether/acyl-linked compounds independently (36). Inositol depletion induced rapid reductions in both species of PI and PIP and strongly reduced diacyl PIP2 levels, but it had little effect on ether/acyl PIP2. Surprisingly, PIP3 was greatly reduced in the *ino1*^−^ mutant under all conditions, independent of exogenous inositol. Overall, the greater reduction in diacyl phosphoinositides, comprising less than 5% of the inositol phospholipids (36), may be due to the preferential metabolism of these species over that of the ether/acyl derivatives as precursors of inositol phosphates. Alternatively, these compounds may provide a more labile signaling component, giving rise to more rapid metabolism than that of ether-derived compounds, and further research could investigate these alternatives. Nevertheless, these data show a critical effect of inositol depletion in regulating phosphoinositides.

These results also suggest an important role of inositol depletion, through regulating diacyl PIP2 in vesicle formation and transport (59) and in membrane trafficking at the neuronal synapse (60). In Dictyostelium, ablation of the PIP2 biosynthetic enzyme PIP5 kinase (PikI) led to a 90% reduction in PIP2 levels and to disoriented cell movement (61). The pivotal role of PIP2 in these processes suggests a requirement for cells to maintain the level of this essential molecule during inositol starvation. Cytokinesis is also critically dependent upon an increase in PIP2 levels (48), and a 65% reduction in diacyl PIP2 levels following 24 h of inositol depletion is consistent with a block in cytokinesis giving rise to the multinucleate phenotype of *ino1*^−^ cells. In a similar manner, PIP2 is involved in substrate attachment by regulating actin polymerization and depolymerization (48), which may result in reduced cell-substrate adhesion. Overall, the data suggest that inositol depletion has a fundamental and rapid effect on phosphoinositide regulation that is likely to result in wide-ranging changes in cellular function and cell health.

Interestingly, Ino1 may play a role in regulating PIP3 levels regardless of the inositol level, since the *ino1*^−^ mutant grown in inositol-supplemented medium showed reduced PIP3 levels, even though intracellular *myo*-inositol levels were comparable to those of wild-type cells. Previous studies in Dictyostelium demonstrated that a complete block of PIP3 production by deletion of all five phosphoinositide 3-kinase enzymes resulted in poor growth and developmental defects (62). Combined, these findings suggest that loss of the Ino1 protein leads to a loss of PIP3 production, resulting in poor cell growth.

We also examined metabolic changes caused by loss of the Ino1 protein and during inositol depletion. Surprisingly, the greatest metabolic change observed here was due to an absence of Ino1, which gave rise to elevated amino acids, energy-related components, DNA regulation, and osmolytes. This metabolic shift was not due to altered inositol levels *per se*, since cellular inositol levels were consistent between the mutant and wild-type cells during inositol supplementation, but rather to an absence of the Ino1 protein. These changes are likely to have a major effect on cellular function and suggest an important noncatalytic role for the protein in metabolic regulation, shifting the metabolism toward an autophagic response, with increased levels of putrescine, amino acids, and nucleotide derivatives (63). In contrast, inositol depletion caused general changes in lipids and variable changes in a few amino acids. This suggests that inositol depletion has little metabolic effect over the short time scale examined in this study.

Since inositol supplementation did not fully restore *ino1*^−^ growth, we expressed the mutant protein Ino1-D342A in these cells and assessed growth. Mutation of this amino acid is likely to disrupt catalytic activity as it is conserved in all known Ino1 proteins. Expression of Ino1-D342A did not rescue the inositol auxotrophy resulting from Ino1 loss, and thus it does not catalyze inositol biosynthesis. In contrast, expression of the protein reduced growth of all strains, independently of exogenous inositol provision. Further studies will be necessary to determine if this response is due to depletion of the Ino1 substrate, inactivation of a potential Ino1 multimeric complex, or other mechanisms.

To identify new roles for Ino1 in the regulation of cellular function, we isolated a number of potential Ino1 binding partners. These included proteins related to cytoskeletal organization, mitochondrial function, DNA and protein regulation, and metabolism, including fatty acid metabolism, glycolysis, and purine metabolism, as well as the vacuolar ATPase, consistent with those identified in S. cerevisiae (64–66) and in humans (67). From the list of potential binding partners, we independently confirmed Ino1-GpmA binding; GpmA catalyzes the production of 2,3BPG from 2- or 3-phosphoglycerate. Importantly, 2,3BPG potently inhibits the dephosphorylation of InsP_3_ and InsP_2_ (68), relating to the effect of lithium on the dephosphorylation of IP_1_ (9), and is elevated following *ino1* loss in Dictyostelium (30). The binding of Ino1-GpmA thus provides a potentially crucial link between loss of Ino1 and a mechanism of inositol depletion similar to that for lithium. We also confirmed strong Ino1-Q54IX5 binding; the Q54IX5 protein contains a tetratricopeptide repeat (TPR) that mediates protein-protein interactions, often during the assembly of multiprotein complexes (69). Although the function of an Ino1-Q54IX5 interaction remains to be examined, the potential human orthologue of Q54IX5 is the SEL1L protein, which is involved in the movement of misfolded proteins from the endoplasmic reticulum to the cytosol and in protein ubiquitination (70), and thus dysregulation of this protein in the *ino1*^−^ mutant may have far-reaching effects on cell function.

Since we showed that the absence of Ino1 and inositol depletion have different molecular and metabolic effects, we questioned whether these effects are interrelated. Inositol depletion has been shown to activate *ino1* expression in a wide range of models (71, 72), including Dictyostelium (5) and mice (73); this effect is likely to elevate Ino1 levels. Many studies have relied on using inositol-depleting drugs prescribed as bipolar disorder treatments, which act through multiple targets (74–77), and hence these studies are likely to be complicated by secondary effects. In contrast, our study did not utilize drug treatments, and our results suggest that short-term inositol depletion does not cause large metabolic changes in Dictyostelium, enabling a subsequent increase in *ino1* transcription to reverse this deficit (5). This responsive regulation would protect cells against a transient reduction in inositol levels without triggering large metabolic changes, with a dysregulation of this responsive mode resulting from a reduction of Ino1 levels causing wide-ranging metabolic effects.

To sum up, our studies show that a loss of the crucial inositol biosynthetic enzyme Ino1 and inositol depletion cause discrete cellular, molecular, and metabolic effects. Although inositol depletion alters cell physiology, triggering an autophagic response, loss of substrate adhesion, reduction in cell division, and rapid reductions in a range of phospholipids, it does not trigger a large change in metabolic profile. In contrast, the Ino1 protein itself plays important roles in cell growth and shape and in metabolic regulation, regardless of inositol level, including binding to a linker protein, Q54IX5, suggesting further roles of this protein.

## Supplementary Material

Supplemental material
