# Supplementary material for: The Inositol-3-Phosphate Synthase Biosynthetic Enzyme Has Distinct Catalytic and Metabolic Roles
Source: Mol Cell Biol. 2016 May 2;36(10):1464–79. doi: 10.1128/MCB.00039-16 (PMC4859692; doi:10.1128/MCB.00039-16)
Supplement: Supplemental material [file supp_36_10_1464__index.html]

The Inositol-3-Phosphate Synthase Biosynthetic Enzyme Has Distinct Catalytic and Metabolic Roles — Supplemental material 

# The Inositol-3-Phosphate Synthase Biosynthetic Enzyme Has Distinct Catalytic and Metabolic Roles

## Supplemental material

- Supplemental file 1 -

  Data Set S1 (Potential binding partners identified by immunoprecipitation)

  XLSX, 21K
